# Discovery of a small arterivirus gene that overlaps the GP5 coding sequence and is important for virus production

**DOI:** 10.1099/vir.0.029264-0

**Published:** 2011-05

**Authors:** Andrew E. Firth, Jessika C. Zevenhoven-Dobbe, Norma M. Wills, Yun Young Go, Udeni B. R. Balasuriya, John F. Atkins, Eric J. Snijder, Clara C. Posthuma

**Affiliations:** 1 Department of Pathology, University of Cambridge, Cambridge, UK; 2 Molecular Virology Laboratory, Department of Medical Microbiology, Center of Infectious Diseases, Leiden University Medical Center, Leiden, The Netherlands; 3 Department of Human Genetics, University of Utah, Salt Lake City, UT 84112-5330, USA; 4 Department of Veterinary Science, Maxwell H. Gluck Equine Research Center, University of Kentucky, Lexington, KY 40546-0099, USA; 5 BioSciences Institute, University College Cork, Cork, Ireland

## Abstract

The arterivirus family (order *Nidovirales*) of single-stranded, positive-sense RNA viruses includes porcine respiratory and reproductive syndrome virus and equine arteritis virus (EAV). Their replicative enzymes are translated from their genomic RNA, while their seven structural proteins are encoded by a set of small, partially overlapping genes in the genomic 3′-proximal region. The latter are expressed via synthesis of a set of subgenomic mRNAs that, in general, are functionally monocistronic (except for a bicistronic mRNA encoding the E and GP2 proteins). ORF5, which encodes the major glycoprotein GP5, has been used extensively for phylogenetic analyses. However, an in-depth computational analysis now reveals the arterivirus-wide conservation of an additional AUG-initiated ORF, here termed ORF5a, that overlaps the 5′ end of ORF5. The pattern of substitutions across sequence alignments indicated that ORF5a is subject to functional constraints at the amino acid level, while an analysis of substitutions at synonymous sites in ORF5 revealed a greatly reduced frequency of substitution in the portion of ORF5 that is overlapped by ORF5a. The 43–64 aa ORF5a protein and GP5 are probably expressed from the same subgenomic mRNA, via a translation initiation mechanism involving leaky ribosomal scanning. Inactivation of ORF5a expression by reverse genetics yielded a severely crippled EAV mutant, which displayed lower titres and a tiny plaque phenotype. These defects, which could be partially complemented in ORF5a-expressing cells, indicate that the novel protein, which may be the eighth structural protein of arteriviruses, is expressed and important for arterivirus infection.

## Introduction

Arteriviruses are enveloped viruses (approx. 60 nm diameter) with a 13–16 kb positive-sense RNA genome. The family *Arteriviridae* (Snijder & Meulenberg, 1998; Snijder & Spaan, 2006) currently comprises four members: equine arteritis virus (EAV, the family prototype), lactate dehydrogenase-elevating virus (LDV) of mice, porcine reproductive and respiratory syndrome virus (PRRSV) and simian haemorrhagic fever virus (SHFV). The consequences of arterivirus infection can range from an asymptomatic, persistent or acute infection to abortion or lethal haemorrhagic fever (Maclachlan *et al.*, 2007). Three of the four arteriviruses (EAV, LDV and SHFV) were first isolated and characterized approximately 50 years ago. In the late 1980s, two distantly related PRRSV genotypes, European (PRRSV-EU) and North American (PRRSV-NA), sharing a genome-wide mean nucleotide identity of only ~63 % (Allende *et al.*, 1999; Suárez *et al.*, 1996), emerged simultaneously on both sides of the Atlantic (Collins *et al.*, 1992; Wensvoort *et al.*, 1991). Since then, the virus has been causing worldwide epidemics of a previously unknown reproductive and respiratory disease and has become one of the most economically important swine diseases. Recently, a large outbreak of highly virulent PRRSV affected the Asian swine industry, causing considerable economic losses (Zhou & Yang, 2010).

The unification of the previously unclassified members of the arterivirus family was the direct result of sequence analysis of their genomes, which also revealed an intriguing relationship with coronaviruses. Despite striking differences in genome size and virion structure, the genome organization and expression strategy of the two groups were found to be comparable and their key replicative enzymes were postulated to share common ancestry (den Boon *et al.*, 1991). A specific feature of their genome expression strategy is the generation of a nested set of subgenomic (sg) mRNAs to express a variety of genes from the 3′-proximal region of the genome (Pasternak *et al.*, 2006; Sawicki *et al.*, 2007). Arteriviruses and coronaviruses were united in the order *Nidovirales*, which was subsequently expanded by the addition of the roniviruses, toroviruses and bafiniviruses (Gorbalenya *et al.*, 2006).

**Fig. 1.**
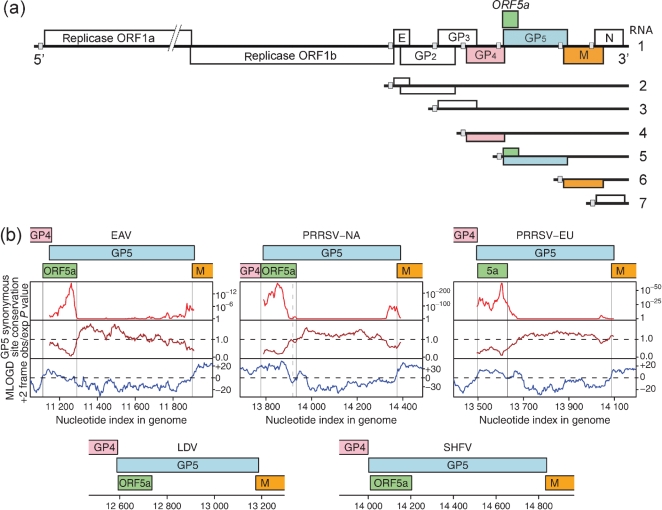
(a) EAV genome organization and expression. The positions of the ORFs encoding the seven known structural proteins and the newly discovered ORF5a are indicated. ORF5a slightly overlaps with the GP4 gene and largely overlaps with the GP5 gene. Small grey boxes indicate the positions of the TRS that direct sg RNA synthesis and serve as junction sequences for the 5′ common leader sequence of arterivirus mRNAs. (b) Maps of the ORF5a/ORF5 region of the genomes of representative arterivirus sequences (see Fig. 2b for GenBank accession numbers). For EAV and the two PRRSV genotypes, plots are shown for several coding-potential statistics in a 15-codon sliding window. The top and middle panels illustrate the degree of conservation at synonymous sites within the GP5 CDS: the top panel depicts the probability that the degree of conservation within a given window could be obtained under a null model of neutral evolution at synonymous sites, while the middle panel depicts the absolute amount of conservation as represented by the ratio of the observed number of substitutions within a given window to the number expected under the null model. Scores below the dashed line correspond to more conserved regions and are indicative of overlapping functional elements, either coding or non-coding. Owing to the huge quantity of GP5 sequence data (8344 sequences) available for the PRRSV-NA plot, the formal *P* values for this analysis are extremely small for the conserved 5′ region. The bottom panel depicts the mlogd coding potential score in the +2 frame (relative to the GP5 CDS). For illustrative purposes the M CDS, and the GP4 CDS in PRRSV-NA, were not included in the null-model CDS annotations so that these CDSs, like ORF5a, register positive coding potential (i.e. scores above the dashed line) in the mlogd analysis. Note that, regardless of sign (positive or negative), the mlogd signal tends to be weaker within regions of high conservation (e.g. within ORF5a) due to there being fewer substitutions with which to discriminate between the null model and the alternative model. A dashed vertical line indicates the 3′ end of the common ORF5a variant (46 codons instead of 51 codons) in PRRSV-NA.

The polycistronic arterivirus genome contains at least nine known ORFs (Fig. 1a). The replicative enzymes are encoded by the 5′-proximal replicase ORFs 1a and 1b, yielding two long non-structural polyproteins, pp1a and pp1ab, with the synthesis of the latter depending on a ribosomal frameshift near the 3′ end of ORF1a. The pp1a and pp1ab precursors are processed into at least 13 mature non-structural proteins (nsps) by three or four viral proteinases encoded by ORF1a (Ziebuhr *et al.*, 2000; Fang & Snijder, 2010). In EAV, PRRSV and LDV, seven relatively small genes have been identified in the 3′-proximal domain of the genome, positioned in the same relative order downstream of ORF1b (Fig. 1a). In SHFV, this region of the genome appears to contain three additional ORFs, which may have arisen from the duplication of ORFs 2–4 (Godeny *et al.*, 1998) but have not been characterized in any detail. In the other arteriviruses, ORFs 2a–7 encode seven structural proteins: the nucleocapsid (N) protein and six envelope proteins, the glycoproteins (GP) GP2, GP3, GP4 and GP5, and the non-glycosylated envelope (E) and membrane (M) proteins (Snijder & Meulenberg, 1998; Snijder & Spaan, 2006). By using reverse genetics to engineer gene knockouts, in both EAV and PRRSV the expression of each of these seven proteins was found to be critical for the production of infectious progeny (Molenkamp *et al.*, 2000; Wissink *et al.*, 2005). These experiments also revealed that cells transfected with mutants lacking expression of E, GP2, GP3 or GP4 release virus-like particles (VLPs) consisting of RNA and the GP5, M and N proteins (Wieringa *et al.*, 2004; Zevenhoven-Dobbe *et al.*, 2004). Together with the earlier characterization of arterivirus particles (de Vries *et al.*, 1992; Faaberg & Plagemann, 1995; Meulenberg *et al.*, 1995; Snijder *et al.*, 1999, 2003; Wieringa *et al.*, 2004), these data indicate that the N protein and the disulphide-linked GP5–M heterodimer form the structural core of the virion. The E protein and a GP2–GP3–GP4 heterotrimer are minor virus constituents that may play a role in receptor binding and/or virus entry (Van Breedam *et al.*, 2010). Still, many details of arterivirus assembly, maturation and structural protein function remain to be elucidated.

The genome organization of the arterivirus ORF2–7 region is extremely compact, with the termini of most ORFs overlapping with neighbouring genes and with RNA signals (transcription-regulatory sequences, TRSs) for sg RNA synthesis commonly being embedded in upstream ORFs (Fig. 1a). These TRSs are assumed to operate as attenuators of negative-sense RNA synthesis and to direct base pairing of the nascent negative-sense strand with a complementary sequence in the genomic 5′ end (leader TRS). This results in the generation of a 3′- and 5′-coterminal set of negative-sense templates that serve to produce the arterivirus sg mRNAs (Pasternak *et al.*, 2006; Sawicki *et al.*, 2007).

The arterivirus genome organization illustrates the strong selective pressure that RNA virus genomes are under to compress the maximum amount of coding and regulatory information into minimal sequence space. Overlapping coding sequences (CDSs) are particularly common in RNA viruses, but can be difficult to detect, especially when they are short. We have been using a number of complementary comparative computational approaches to systematically identify novel overlapping genes in RNA virus genomes (Firth & Brown, 2006; Chung *et al.*, 2008; Firth *et al.*, 2008; Firth & Atkins, 2009). When applied to arteriviruses, these methods detected a novel short ORF, hereafter termed ORF5a, overlapping the 5′ end of the GP5 CDS in the +2 reading frame. Here, we present bioinformatic evidence and experimental verification for the functional importance of ORF5a in arteriviruses.

## Results and Discussion

### Computational analysis reveals the presence of a small ORF overlapping the arterivirus GP5 gene

**Fig. 2.**
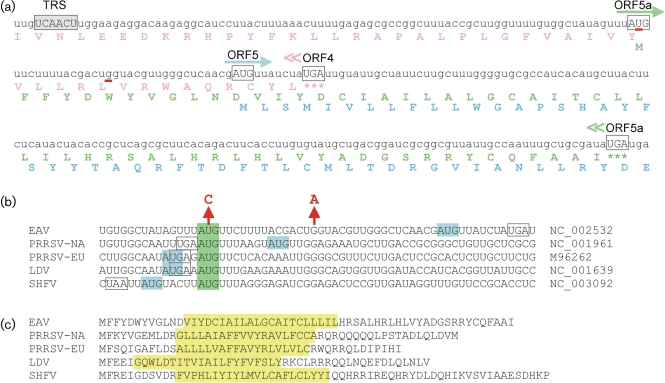
Expression of arterivirus ORF5a. (a) RNA sequence and translation of the EAV ORF5a region, showing the slightly overlapping GP4 and GP5 genes, and the newly discovered ORF5a that overlaps with both. The TRS for sg RNA5 synthesis and relevant initiation and termination codons are indicated. Red underscores indicate residues mutated to knock out ORF5a in mutant Δ5a-2. (b) Context of the ORF5a (green) and ORF5 (blue) translation initiation codons in five representative arterivirus sequences, illustrating the potential for leaky scanning being the translation initiation mechanism for the second reading frame (see text for details). The figure shows that the ORF5a initiation codon is the more upstream in EAV and PRRSV-NA and the more downstream in the other arteriviruses. The ORF4 termination codon is boxed and the two residues mutated to knock out EAV ORF5a in mutant Δ5a-2 are indicated by arrows. (c) Amino acid sequences of the ORF5a proteins of five representative arterivirus sequences. The position of the putative transmembrane domain, as predicted by phobius (http://phobius.binf.ku.dk; Käll *et al.*, 2004), is indicated in yellow. The ORF5a protein is predicted to be a type III membrane protein (lumenal amino-terminal domain, central signal-anchor/transmembrane domain and cytoplasmic carboxy-terminal domain).

The coding potential of ORF5a was first detected in an alignment of PRRSV-NA sequences by using the gene-finding software mlogd (Firth & Brown, 2006; Chung *et al.*, 2008). mlogd uses nucleotide and amino acid substitution matrices to model sequence evolution in dual-coding, single-coding and non-coding regions. It can be used to predict whether query ORFs are likely to be coding sequences via an approximate likelihood-ratio test, where the null model comprises any known CDSs and the alternative model comprises the known CDSs and the query ORF. When applied to the PRRSV-NA alignment, mlogd detected a positive coding signature for the previously defined arterivirus CDSs and also for ORF5a (Fig. 1b). In the National Center for Biotechnology Information (NCBI) PRRSV-NA reference sequence, NC_001961 (15 428 nt), ORF5a covers nucleotides 13 778–13 930 (51 codons) and therefore overlaps the 5′ end of the GP5 CDS (nucleotides 13 788–14 390) by 48 codons (~24 % of the GP5 gene) in the +2 reading frame. ORF5a begins immediately 3′ of the ORF4 termination codon and 10 nt 5′ of the ORF5 initiation codon (Fig. 2b).

A similar ORF5a was identified in PRRSV-EU and the other arteriviruses, EAV (Fig. 2a), LDV and SHFV, and again mlogd detected a positive coding signature for ORF5a using sequence alignments of PRRSV-EU, EAV and LDV. Incidentally, the presence of this ORF in EAV, though not its bioinformatic coding signature, had previously been noted by Pasternak *et al.* (2000). Since there is only a single SHFV sequence available, mlogd could not be used to test the coding potential of ORF5a in that virus. Although this analysis was based on the application of mlogd to specific ORFs, for illustrative purposes the mlogd score in a 15-codon sliding window in the +2 reading frame (with respect to ORF5) is shown in Fig. 1(b) for alignments of 127 EAV, 232 PRRSV-NA and 34 PRRSV-EU sequences (i.e. the available sequences with coverage of both ORF5 and at least 150 nt of its 5′ and 3′ flanking sequences). Positive coding signatures are apparent for ORF5a (and also for the M and GP4 CDSs in cases where they fall in the +2 reading frame with respect to the GP5 CDS). Additional alignments were generated for 224 EAV, 8344 PRRSV-NA and 587 PRRSV-EU sequences that have full coverage of the GP5 CDS, and were analysed for conservation at synonymous sites as described in Firth & Atkins (2009) [see also Simmonds *et al.* (2008); see Shi *et al.* (2010) and Zhang *et al.* (2010) for recent PRRSV and EAV phylogenies]. Enhanced conservation at synonymous sites is indicative of overlapping functional elements – either coding or non-coding. In all three alignments, greatly enhanced conservation was apparent in the region where ORF5a overlaps the GP5 CDS (Fig. 1b).

The newly discovered ORF5a has 59 codons in EAV, either 51 or 46 codons in PRRSV-NA, 43 codons in PRRSV-EU, 47 codons in LDV and 64 codons in SHFV (Fig. 2c). When applied to the five representative sequences in Fig. 2(c), despite there being considerable sequence divergence, phobius and signalp 3.0 (Käll *et al.*, 2004; Bendtsen *et al.*, 2004) consistently predict that the ORF5a protein is a type III membrane protein with a short (5–12 aa) lumenal domain at its amino terminus, a central signal-anchor/transmembrane sequence, and a cytosolic carboxy-terminal domain of 13–31 aa.

### The ORF5a protein and GP5 are probably expressed from the same subgenomic mRNA via leaky scanning

The regions around the ORF5a initiation codon in five representative arterivirus sequences are shown in Fig. 2(b). In EAV and PRRSV-NA, the ORF5a AUG codon is 5′ of the ORF5 initiation codon and the TRS for the sg RNA5 is located sufficiently far upstream to include the ORF5a AUG codon within that sg mRNA, with no additional intervening AUG codons (Snijder & Meulenberg, 1998; Pasternak *et al.*, 2000; Lin *et al.*, 2002). On the other hand, in PRRSV-EU, LDV and SHFV, the ORF5a AUG codon is located just a few nucleotides 3′ of the ORF5 initiation codon.

In eukaryotes, the efficiency of translation initiation is modulated by the identity of the nucleotides surrounding the initiation codon, with the optimal context (known as the ‘Kozak consensus sequence’) in mammals involving mainly a guanine at the +4 position and a purine at the −3 position, where the presence of an adenine at the −3 position is the strongest single indicator of efficient initiation (Kozak, 2002). When the context of the first AUG codon on a transcript is suboptimal, efficient leaky scanning to alternative downstream AUG codons can occur (Kozak, 2002). Here, neither the ORF5 nor ORF5a initiation codons have a guanine at the +4 position, but all five arterivirus ORF5 initiation codons have an adenine at the −3 position. In contrast, the ORF5a initiation codons have either a pyrimidine (EAV, PRRSV-NA and SHFV) or a guanine (PRRSV-EU and LDV) at the −3 position. Thus, in PRRSV-NA and EAV, where the ORF5a AUG codon is 5′, there is the potential for efficient leaky scanning to the ORF5 initiation codon. In contrast, in PRRSV-EU, LDV and SHFV, where the ORF5a AUG is 3′, conventional leaky scanning for ORF5a translation may be expected to be less efficient. However, in these sequences, the ORF5 and ORF5a AUG codons are very closely spaced (separated by just 2 or 5 nt) and it has been demonstrated that, if two AUG codons are separated by less than ~10 nt, efficient initiation may occur at the downstream AUG codon, even when the upstream AUG codon is in a strong context (Matsuda & Dreher, 2006).

The analysis of initiation codons and their context, and ORF5a length, was extended to all available arterivirus sequences in GenBank. Some 10 800 sequences with full or partial coverage of the ORF5a/ORF5 region were analysed. In contrast to the above comparative phylogenetic analyses, simply counting sequences ignores potential phylogenetic bias (some clades may be greatly over-represented relative to others; indeed; clustering the sequences with blastclust or clustal x shows this to be the case). Thus, the following analysis is more meaningful with respect to the range of features present rather than to the relative abundances of those features. Many sequences (including ~90 % of the ~9600 available PRRSV-NA sequences) cover ORF5 entirely but have no 5′ flanking sequence. Therefore they do not allow analysis of the upstream ORF5a initiation codon or its context, but still allow analysis of the presence or absence of premature termination codons within the part of ORF5a that overlaps ORF5. Nonetheless 136 EAV, 445 PRRSV-NA, 622 PRRSV-EU, 1 SHFV and 5 LDV sequences have coverage of the ORF5a initiation site, and the above-mentioned initiation sites and contexts are very highly conserved (except that ~63 % of PRRSV-EU and ~9 % of PRRSV-NA sequences have a guanine instead of an adenine as the nucleotide at the −3 position of the ORF5 initiation codon context). Similarly, the above-mentioned ORF5a lengths are highly conserved in those sequences with full or partial coverage of the ORF5a region, with the notable exception that PRRSV-NA exhibits two main forms of differing lengths: a 51 codon version (~26 % of sequences) and a 3′-truncated 46-codon version (~74 % of sequences). A very small fraction of sequences were found to be ORF5a-defective as a result of either premature ORF5a-frame termination codons or substitutions within the ORF5a initiation codon. However, these do not necessarily represent sequences from viable viruses, but might instead be due to sequencing errors, sequencing of defective virus sequences or curation errors (e.g. five sequence records in which non-contiguous GP3 and GP5 sequences appear to have been fused *in silico*). Indeed, similar numbers of GP5-defective sequences were found during the analysis.

To summarize, four completely separate computational analyses – conservation of an ORF, conservation of a potential translation mechanism, positive mlogd coding signature and a greatly suppressed frequency of substitutions at ORF5-frame synonymous sites – all pointed to the existence of a novel coding sequence, ORF5a.

### Inactivation of EAV ORF5a expression dramatically affects virus production

To investigate the biological relevance of ORF5a in the arterivirus replicative cycle, we used an EAV reverse-genetics system (van Dinten *et al.*, 1997) to engineer two virus mutants in which ORF5a expression was inactivated. In both mutants, Δ5a-1 and Δ5a-2, the ORF5a translation initiation codon was mutated (AUG to ACG; U-11113→C), while in the second mutant a premature termination codon was engineered at codon six of ORF5a (G-11128→A). Care was taken that the mutations introduced were translationally silent with respect to the overlapping GP4 gene (Fig. 2b). Following electroporation of *in vitro*-synthesized full-length RNA into BHK-21 cells, which are also susceptible to EAV infection, the phenotype of the ORF5a knockout mutants was compared with that of the wild-type control. Dual-labelling immunofluorescence (IF) microscopy for replicase (nsp3) and N protein was performed to verify comparable transfection efficiency and monitor the synthesis of genome and subgenomic mRNA7, respectively (not shown). Viral RNA synthesis in transfected cells was also analysed by Northern blot hybridization (not shown). Finally, infectivity titres in the medium of transfected cells were determined by plaque assay, both after the first cycle (18 h post-transfection) and following subsequent virus amplification in initially non-transfected BHK-21 cells.

**Fig. 3.**
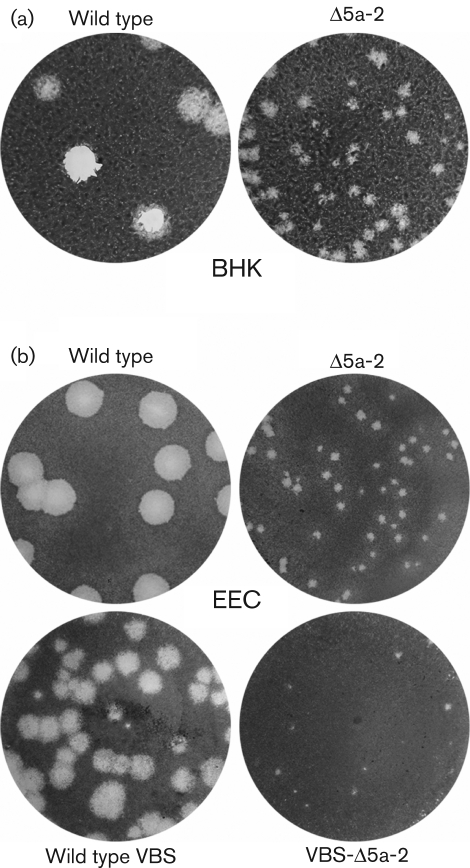
(a) Comparison of plaque morphology on BHK-21 cells of wild-type EAV and ORF5a knockout mutant Δ5a-2, illustrating the severely crippled phenotype of the latter. In addition to the dramatically reduced plaque size, Δ5a-2 infectivity titres were reduced by approximately two logs. (b) Comparison of wild-type and Δ5a-2 plaque morphology on equine endothelial (EEC) cells using both a cell-culture-adapted EAV isolate (EAV515) and an isolate that is virulent in horses (VBS). In both virus backbones, Δ5a-2 plaque sizes and titres are severely reduced compared with those of the corresponding parental virus.

Both IF microscopy and hybridization analysis of intracellular viral RNA demonstrated that the two ORF5a knockout mutants were viable and that their RNA synthesis could not be distinguished from that of the wild-type control. However, the analysis of viral progeny released into the medium of transfected cells revealed that both ORF5a knockout mutants were seriously crippled. Compared with the wild-type virus, they showed a reduction in virus titre of approximately two logs, and a tiny plaque size (Fig. 3a). Upon analysis of the 40 h post-transfection harvest of mutant Δ5a-1, several large plaques were also observed, suggesting rapid reversion of its single point mutation to the wild-type sequence, which was subsequently confirmed by sequence analysis of the ORF5a region of the progeny virus. However, no reversion was observed for mutant Δ5a-2, in which a double point mutation had been engineered.

### Confirmation of the importance of EAV ORF5a in a second, virulent EAV isolate and in natural host cells

EAV strains can differ significantly in the severity of the disease they induce in their natural host (Balasuriya *et al.*, 2007; Maclachlan *et al.*, 2007). The molecular clone used in the initial experiments described above (pEAV515, a derivative of pEAV030; van Dinten *et al.*, 1997) was based on an EAV isolate that was extensively adapted to cell culture (>300 passages) and causes very mild clinical signs upon experimental infection of horses (Balasuriya *et al.*, 1999). To independently confirm the effect of an ORF5a knockout in a second EAV isolate, the two ORF5a-inactivating nucleotide substitutions of mutant Δ5a-2 were transferred to a molecular clone (pEAVrVBS) of the virulent Bucyrus strain (VBS) of EAV, which can cause severe disease in horses (Balasuriya *et al.*, 2007). Furthermore, in order to use a more natural host cell than the BHK-21 cells used above, we transfected the *in vitro*-synthesized full-length RNAs into equine pulmonary artery endothelial cells (EEC; Hedges *et al.*, 2001). Again, in both virus backbones, the Δ5a-2 mutant displayed a severely crippled phenotype, with tiny plaques (Fig. 3b) and infectivity titres reduced by at least two logs. Thus, this comparative study independently confirmed that the expression of ORF5a is critical for efficient EAV amplification, irrespective of the background of the molecular clone and cell type used for analysis.

### Partial complementation of the EAV ORF5a knockout defect in ORF5a-expressing cells

Despite the overwhelming bioinformatic evidence for the functionality of ORF5a, we wanted to exclude the possibility that the crippled phenotype of the Δ5a-2 mutant was due to damaging some unidentified yet important RNA signal in the EAV genome. To this end, we investigated whether the infectivity defect could be complemented in a cell line expressing the ORF5a protein. In view of previous success in rescuing EAV structural-protein knockouts in BHK-21 cells containing alphavirus RNA replicons (Zevenhoven-Dobbe *et al.*, 2004), ORF5a was cloned into pSinRep19, a Sindbis virus (SINV)-based expression vector (Agapov *et al.*, 1998). BHK-21 cells were transfected with the EAV ORF5a-expressing SinRep19 replicon and positive selection was applied by adding puromycin to the culture medium. BHK-21 cells containing a SinRep19 replicon that expresses GFP (SinRep19–GFP; Agapov *et al.*, 1998; Zevenhoven-Dobbe *et al.*, 2004) were used as a negative control.

**Fig. 4.**
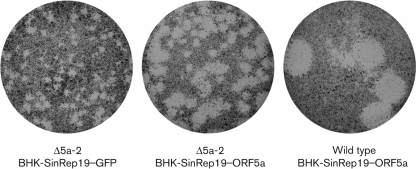
Complementation of the EAV ORF5a knockout mutant Δ5a-2 in ORF5a-expressing cells. The Δ5a-2 small-plaque phenotype (left) was partially complemented when the plaque assay was performed using BHK-21 cells carrying an ORF5a-expressing SINV RNA replicon (middle; Agapov *et al.*, 1998). As a negative control, cells carrying a GFP-expressing replicon were used. The plaque morphology of wild-type virus on ORF5a-expressing cells is shown on the right.

Plaque assays of wild-type and Δ5a-2 virus were performed in both the ORF5a- and GFP-expressing cell cultures. While no difference in plaque size was observed for wild-type virus (not shown), the Δ5a-2 virus plaque size was clearly increased in ORF5a-expressing cell cultures (Fig. 4). A growth curve experiment in which GFP- and ORF5a-expressing cells were infected with Δ5a-2 mutant virus showed that the Δ5a-2 titre increased up to fivefold in the ORF5a-expressing cells (data not shown). Complete complementation to wild-type plaque size and titre could not be achieved, which may be due to a variety of technical or intrinsic factors such as expression level, but these experiments clearly demonstrated that the expression of the ORF5a protein directly influences the EAV life cycle.

### The ORF5a protein: the eighth structural protein of arteriviruses?

In this study, we present evidence for the presence, conservation and biological significance of a previously unrevealed short CDS in the 3′-proximal domain of all arterivirus genomes. Much like the case of the arterivirus E and GP2 genes, whose translation is assumed to be initiated following leaky scanning of the 5′-proximal domain of sg mRNA2 (Snijder *et al.*, 1999), ORF5a and the overlapping GP5 gene are also likely to be expressed from a functionally bicistronic sg mRNA. Another striking similarity with the E–GP2 gene pair (Snijder *et al.*, 1999) is the fact that the ORF5a initiation codon precedes the GP5 gene in some arteriviruses (EAV and PRRSV-NA), but is just downstream of the ORF5 initiation codon in the others. This observation raises interesting questions about the evolution of this part of the arterivirus genome, in particular when evolving optimal sg mRNA synthesis and the expression levels of each of the viral structural proteins are taken into account. In EAV, a 10 nt segment starting with the ORF5 initiation codon can now be assumed to encode amino acids for three different proteins, GP4, the ORF5a protein and GP5, in three different reading frames, which again illustrates the pressure on RNA viruses to compress maximal coding information into a minimal sequence.

Previous attempts to engineer EAV-based vectors might, unknowingly, have been affected by the presence of ORF5a. For example, in one construct the overlap between ORF4 and ORF5 was removed to create an insertion site for foreign sequences (de Vries *et al.*, 2000). This was achieved by using a 24 nt insertion that, fortuitously, now proves to be exactly in-frame with ORF5a, although in the ORF5a protein it introduces the substitution of Asp-12 for Ala and Tyr-15 with the sequence Cys-Glu-Leu-Lys-Gln-His-Ala-Val-His. In view of the reported wild-type phenotype of this recombinant virus (de Vries *et al.*, 2000), this drastic change appears to be tolerated. Similarly, after passaging in cell culture 70 times, a spontaneous insertion of 12 nt into ORF5a (Ala-Arg-Ser-Leu inserted after the amino-terminal Met-Phe) of PRRSV-NA was tolerated (Han *et al.*, 2009). In contrast, derivatives of the EAV-based vector in which the 24 nt-sequence served to insert additional foreign sequences, encoding e.g. GFP (de Vries *et al.*, 2001) or ectodomains of other arterivirus GP5 proteins (Dobbe *et al.*, 2001), must have suffered because of the disruption of ORF5a. In fact, the instability and crippled or non-viable phenotypes reported for some of these constructs could (in part) be explained by a lack of expression of a functional ORF5a protein.

The lack of sequence similarity with the contents of protein databases, the protein sequence plasticity described above and the limited sequence conservation among arteriviruses (Fig. 2c) make it difficult to postulate a function for the ORF5a protein. Its expression from the 3′-proximal domain of the genome, and the apparent lack of an effect of ORF5a inactivation on viral RNA synthesis, would suggest that the protein is an additional virion component, and its hydrophobicity profile would place it in the virus envelope. On the other hand, knockouts of the other seven structural-protein genes were lethal to the virus, suggesting that the ORF5a protein belongs to a different functional category. Unfortunately, several attempts to raise antisera using synthetic peptides representing sequences from the non-hydrophobic domain of the EAV ORF5a protein were unsuccessful, thus obstructing our efforts to directly analyse virus particles for the presence of the ORF5a protein or analyse its subcellular localization in the infected cell. We used the SINV replicon system to express ORF5a proteins with epitope tags (haemagglutinin- or myc-tags at either the amino or carboxy terminus), but the Δ5a-2 mutant virus was no longer complemented by these fusion proteins. Finally, we were unable to demonstrate the presence of the ORF5a protein in virions by mass spectrometric analysis of purified EAV. However, a recent proteomics analysis of purified PRRSV-NA did identify several peptides from the ORF5a protein of the porcine arterivirus, and follow-up studies confirmed both the expression of the novel protein in infected cells and the production of antibodies recognizing the PRRSV ORF5a protein in infected swine (Johnson *et al.*, 2011). Thus, the combined data now obtained for PRRSV-NA and EAV strongly suggest that the ORF5a protein is the eighth structural protein of the arterivirus particle, although more extensive studies will be required to gain detailed insight into its function in arterivirus biology.

## Methods

### 

#### Computational analysis.

Virus sequences were obtained from GenBank initially in Feb 2007 and again in Nov 2010. Arterivirus nucleotide sequences with coverage of the ORF5 region were identified by applying NCBI tblastn (Altschul *et al.*, 1990) to the GP5 amino acid sequences from the five reference sequences shown in Fig. 2(b). In total one SHFV, five LDV, 435 EAV and 10 359 PRRSV sequences with full or partial coverage of ORF5 were retrieved. PRRSV sequences were separated into PRRSV-NA and PRRSV-EU genotypes (with respect to GP5), based on the tblastn results. Using a 76 % cut-off threshold on the tblastn ‘maximum identity' score, all but eight of the 10 359 sequences were cleanly separated between the two genotypes. Within each group, ORF5 sequences were translated, aligned and back-translated to nucleotide sequence alignments using emboss and clustal (Rice *et al.*, 2000; Larkin *et al.*, 2007). Flanking nucleotide sequences, where available, were similarly extracted, aligned and combined into the alignments. Patent sequences and constructs were removed prior to analysis, and sequences annotated as defective (found by using the search terms ‘non-functional', ‘truncated' and ‘defective' in GenBank records) were inspected and removed from the analyses where appropriate. mlogd statistics were calculated as described by Firth & Brown (2006). The synonymous site conservation statistic was calculated as described by Firth & Atkins (2009). Prior to these latter two analyses: (i) sequences with premature termination codons in ORF5 or ORF5a were also removed as being presumed-defective sequences (these amount to <1 % of sequences and are not obviously clustered phylogenetically), (ii) one EAV sequence with nine ambiguous nucleotide codes – indicative of poor sequence quality – was removed and (iii) since there was no shortage of PRRSV-NA sequences, PRRSV-NA sequences with any ambiguous nucleotide codes (~10 % of sequences) were removed.

#### Cells and plaque assays.

EEC cells (Hedges *et al.*, 2001) were maintained in Dulbecco’s modified Eagle’s medium (DMEM; Mediatech) with 110 mg l^-1^ sodium pyruvate, 10 % FBS (HyClone; Thermo Scientific), 100 U penicillin ml^-1^ and 100 µg streptomycin ml^-1^ (Invitrogen), and 200 mM l-glutamine. BHK-21 cells (ATCC CCL10) were grown in Eagle’s minimum essential medium with 10 % ferritin-supplemented bovine calf serum and 100 U penicillin ml^-1^ and 100 µg streptomycin ml^-1^. Plaque assays on BHK-21 cells were performed for 3–5 days as described previously (Nedialkova *et al.*, 2010), whereas EEC cells were overlaid with DMEM containing 0.75 % carboxymethyl cellulose and incubated for 4 days.

#### Engineering of EAV ORF5a knockout mutants and reverse genetics.

ORF5a knockout mutants Δ5a-1 and Δ5a-2 (Fig. 2b) were created as follows: point mutations were introduced in an appropriate shuttle vector by standard site-directed PCR mutagenesis (QuikChange Site-Directed Mutagenesis kit; Agilent Technologies). Using suitable restriction sites, the desired mutations in the ORF5a-coding sequence were transferred into EAV full-length cDNA clone pEAV515 or into pEAVrVBS, an infectious cDNA clone of the virulent Bucyrus strain of EAV (Balasuriya *et al.*, 2007). pEAV515 is a derivative of the EAV cDNA clone pEAV030 (van Dinten *et al.*, 1997), in which the *Bgl*II site at position 5217 in ORF1a was knocked out by using a translationally silent substitution. EAV515 virus has a wild-type phenotype, as determined by comparison with pEAV030-derived virus in growth-curve experiments and plaque assays. BHK-21 or EEC cells were electroporated with *in vitro*-synthesized full-length RNA transcripts of wild-type and mutant cDNA clones as described previously (van Dinten *et al.*, 1997). For IF assays, cells were seeded on coverslips, paraformaldehyde-fixed and double labelled for EAV nsp3 and N protein as described by van der Meer *et al.* (1998). Analysis of revertants was done by infecting fresh BHK-21 cells with supernatant harvested at 40 and 72 h post-transfection. Total RNA was isolated after 24 h by using TRIzol (Invitrogen) and the ORF5a region was amplified by RT-PCR and sequenced using standard protocols.

#### Generation of ORF5a-expressing BHK-21 cells.

The coding sequence of ORF5a was PCR amplified using sense primer E1036 (CGTACGCGTCCATGGGGTTCTTTTACGACTGGTACGTTG; *Mlu*I site underlined) and antisense primer E1037 (CGTGCATGCTCATATCGCAGCAAATTGGCAATA; *Sph*I site underlined), and cloned between the *Mlu*I and *Sph*I sites of the pSinRep19 multiple cloning site (Agapov *et al.*, 1998). To obtain EAV ORF5a-expressing BHK-21 cells (BHK-SinRep19–ORF5a), *in vitro*-transcribed SinRep19–ORF5a RNA was transfected and puromycin selection (5 µg ml^−1^) was applied from 24 h post-transfection onwards. A SinRep19 vector expressing GFP was used to produce a control cell line (BHK-SinRep19–GFP).
